# Supplementary material for: Functionalized Titanium Dioxide Nanoparticle-Based Electrochemical Immunosensor for Detection of SARS-CoV-2 Antibody
Source: Diagnostics (Basel). 2022 Oct 27;12(11):2612. doi: 10.3390/diagnostics12112612 (PMC9689474; doi:10.3390/diagnostics12112612)
Supplement: Supplementary file 1 [file diagnostics-12-02612-s001.zip › diagnostics-1971140-supplementary.pdf]

Supplementary Information

Functionalized Titanium Dioxide Nanoparticle-Based Electrochemical Immunosensor for Detection of SARS-CoV-2 Antibody

Mohd Abubakar Sadique [1,2], Shalu Yadav [1,2], Vedika Khare [3], Raju Khan [1,2,*], Gagan Kant Tripathi [3] and Purnima Swarup Khare [3]

[1] Industrial Waste Utilization, Nano and Biomaterials, CSIR-Advanced Materials and Processes Research Institute (AMPRI), Bhopal 462026, India
[2] Academy of Scientific and Innovative Research (AcSIR), Ghaziabad 201002, India
[3] School of Nanotechnology, Rajiv Gandhi Proudyogiki Vishwavidyalaya, Bhopal 462033, India
* Correspondence: khan.raju@ampri.res.in

Synthesis of TiO_2 Nanoparticles

TiO_2 NPs with a basic medium and the neutral medium was prepared by a similar method as TiO_2 NPs with an acidic medium had been synthesized with some modifications in the method [1]. In brief, an equal volume of ethanol and water was mixed with orthophosphoric acid keeping the pH around 2–3. The above solution mixture was stirred for 5 seconds at ambient temperature. To the above solution, titanium isopropoxide was added as the precursor, and soon after the reaction was halted. The reaction was completed, and the product (precipitate) was filtered and dried out. The resulting substance was manually pulverized into a powder and washed 4 times with DI water at 8,000 rpm, followed by calcination at 500 °C for 2 hours and furnace cooling. TiO_2 NPs with a basic medium and the neutral medium was prepared by a similar method as TiO_2 NPs with an acidic medium had been synthesized with some modifications in the method [1]. Titanium isopropoxide was used as the precursor, 40 ml of each ethanol and water was taken in a beaker, and for maintaining the pH around 11, 0.8 ml of NaOH was added. For 5–10 seconds, components were stirred at 25 °C. Then, 10 ml of titanium isopropoxide was added to the above solution. The stirring was stopped after the addition of titanium isopropoxide. The reaction precipitate was formed and filtered out followed by drying in the oven. The resulting product was manually ground into a powder and washed 4 times with DI water at 8,000 rpm, followed by calcination at 500 °C for 2 hours and furnace cooling. Likewise, TiO_2 NPs with maintaining the pH at a neutral range have been prepared.

X-ray Diffraction of TiO_2 Nanoparticles

The X-ray diffraction patterns of synthesized TiO_2 NPs in the acidic, basic, and neutral media were exposed in **Figure S1**. The XRD outcome of TiO_2 NPs in acidic and neutral media, shown in **Figure S1a** and **Figure S1c** has diffraction peaks at 2θ lues of 25.28°; 38.7°; 48.05°; 53.89°; 55.06°; 62.69°; 68.74°; 70.16° and 75.14° which corresponds to the tetragonal anatase crystalline phase and their matching *hkl* planes (101), (004), (200), (105), (211), (204), (116), (220) and (215) respectively, which agrees with the JCPDS card no. 21–1272 (anatase TiO_2 NPs). This plane displays strong intensity at 25.28° in comparison to all of the other peaks. The X-ray diffraction pattern in **Figure S1b,** shows the broad peaks at 25.64° and 48.79° having corresponding planes (101) and (200) with some background peaks. As shown in **Figure S1d** TiO_2 NPs synthesized using a basic medium showed low crystallinity compared to those synthesized in a neutral and acidic media. The observed pattern also confirms the crystalline nature of the particles.

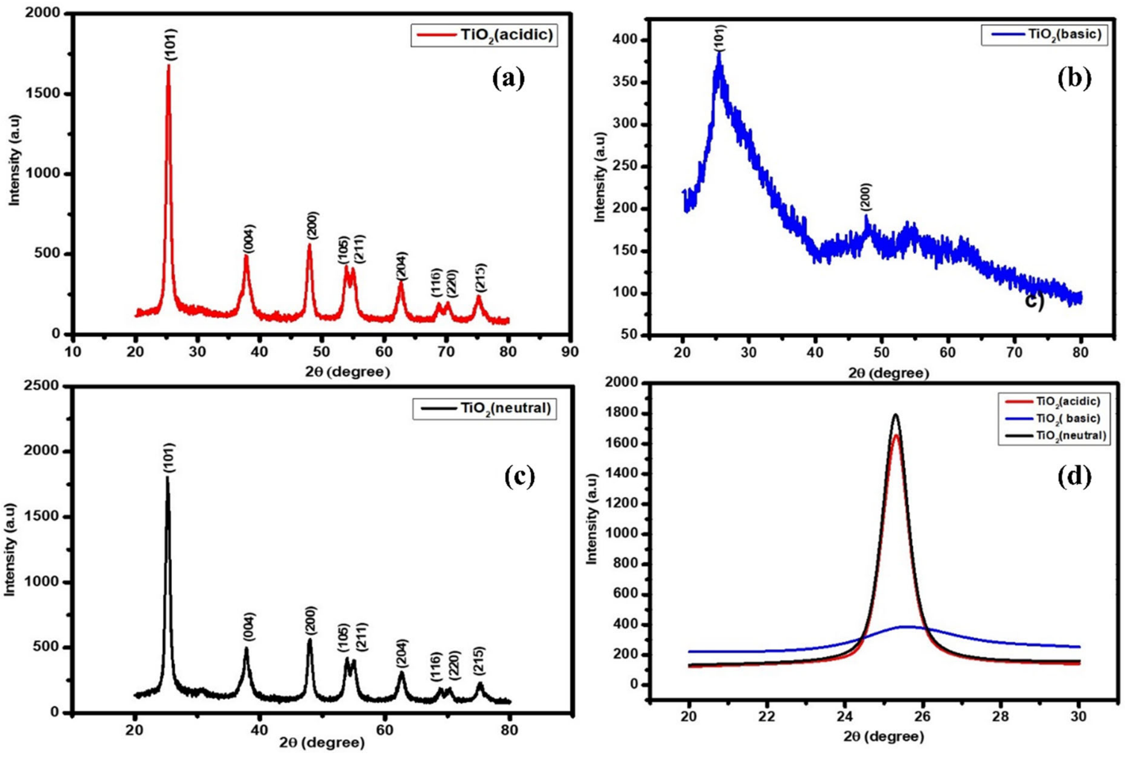

Figure S1. XRD results of TiO_2 NPs in **a)** acidic medium, **b)** basic medium, **c)** neutral medium, and **d)** Crystal phase comparison at 2θ =25.64°.

Table S1. Crystalline size of TiO_2 NPs.

Sr. No.	Nanoparticles (NPs)	FWHM (β)	Diffraction peak (θ)	Cos θ	Crystalline size (D) (nm)
1.	TiO_2 (acidic)	0.753	12.64	0.997	19.7
2.	TiO_2 (basic)	0.353	12.75	0.983	41.72
3.	TiO_2 (neutral)	0.776	12.64	0.997	18.7

Comparative Electrochemical Analysis in Various pH Media

An elaborated electrochemical analysis of TiO_2 NPs and TiO_2-CS bio-nanocomposite synthesized in different pH media has been carried out to compare the conductivity of the NPs. **Figure S2a** and **Figure S3a** show the CV curve of TiO_2 NPs and TiO_2-CS bio-nanocomposite in an acidic medium respectively and have the highest peak current as compared to the bare GCE, TiO_2 NPs, and TiO_2-CS bio-nanocomposite in a basic and neutral media. This pattern has been associated with DPV as well as with EIS results shown in **Figure S2b**, **Figure S2c** and **Figure S3b**, **Figure S3c** respectively.

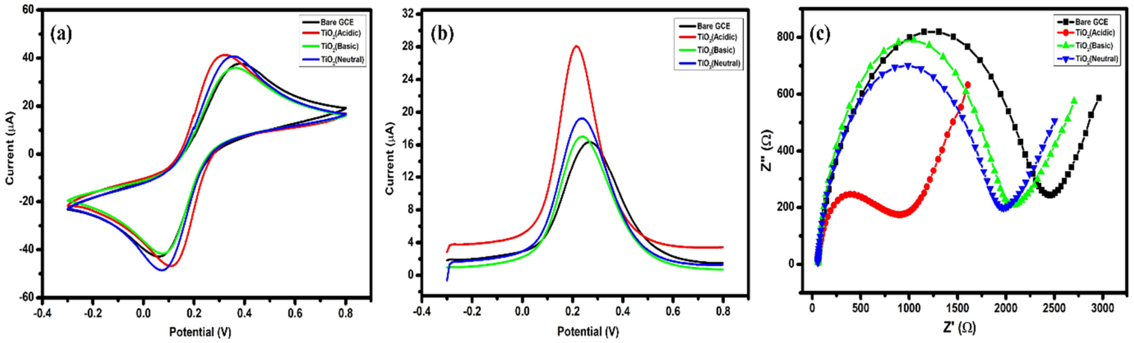

Figure S2. Comparative electrochemical analysis through **a)** CV, **b)** DPV and **c)** EIS of TiO_2 NPs in different pH media.

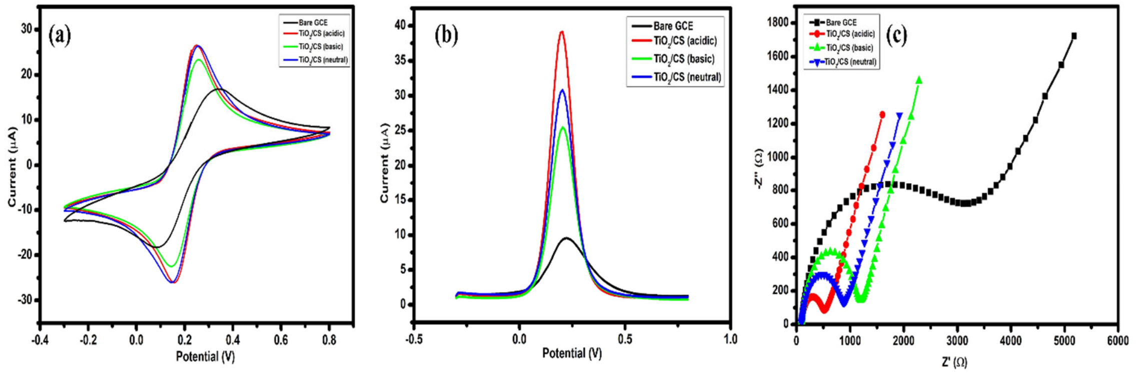

Figure S3: Comparative electrochemical analysis through **a)** CV, **b)** DPV and **c)** EIS of TiO_2-CS bio-nanocomposite in different pH media

Table S2. Ipa, Ipc, Ea, Ec values of corresponding TiO_2 NPs.

Sr. No.	Samples	Anodic peak current I_{pa} (μA)	Anodic potential E_{pa} (V)	Cathodic peak current I_{pc} (μA)	Cathodic potential E_{pc} (V)
1.	Bare GCE	37.66	0.37	42.84	0.06
2.	TiO_2 NPs (acidic)	41.33	0.32	46.78	0.10
3.	TiO_2 NPs (basic)	35.95	0.36	41.65	0.07
4.	TiO_2 NPs (neutral)	40.75	0.35	48.50	0.07

Table S3. Peak current, voltage, and R_{ct} values of TiO_2 NPs.

Sr. No.	Samples	Peak current (μA)	Voltage (V)	R_{ct} (Ω)
1	Bare GCE	16.36	0.26	2454
2.	TiO_2 NPs (acidic)	28.09	0.21	906
3.	TiO_2 NPs (basic)	17.01	0.24	2083
4.	TiO_2 NPs (neutral)	19.24	0.23	1976

Table S4. Summarizing the effective surface area at each step of immunosensor fabrication.

**Modified Layer**	**Peak Anodic current (I_{pa}) (μA)**	**Effective surface area (cm^2)**
GCE/TiO_2	41.20	8.04 ×10^{-2}
GCE/TiO_2-CS	48.29	9.42 ×10^{-2}
GCE/TiO_2-CS/Antigen	40.62	7.92 ×10^{-2}
GCE/TiO_2-CS/Antigen/ BSA	33.29	6.49 ×10^{-2}

Table S5. The polarization resistance (R_p), redox solution resistance (R_s), and charge transfer resistance (R_{ct}) of each surface-modified electrode *via* EIS analysis.

Sr. No.	Electrode	Peak Current (μA)	Potential (V)	R_s (Ω)	R_p (Ω)	R_{ct} (Ω)
1.	Bare GCE	10.66	0.28	0.058	3.420	3.362
2.	GCE/TiO_2	21.29	0.20	0.048	1.515	1.467
3.	GCE/TiO_2-CS	40.67	0.17	0.052	0.766	0.714
4.	GCE/TiO_2-CS/ Antigen	18.25	0.19	0.051	1.844	1.793
5.	GCE/TiO_2-CS/Antigen/BSA	11.37	0.19	0.054	3.085	3.031

References

1. Prakash, M.; Ghosh, A.K. An Investigation on Optimization of Instantaneous Synthesis of TiO2 Nanoparticles and It's Thermal Stability Analysis in PP-TiO2 Nanocomposite. *Solid State Sci* **2021**, *120*, 106707, doi:https://doi.org/10.1016/j.solidstatesciences.2021.106707.
